# Crystal structure of poly[bis­(μ-nicotinamide-κ^2^ *N* ^1^:*O*)bis­(μ-4-nitro­benzoato-κ^2^ *O* ^1^:*O* ^1′^)zinc]

**DOI:** 10.1107/S2056989015006490

**Published:** 2015-04-11

**Authors:** Gülçin Şefiye Aşkın, Hacali Necefoğlu, Ali Murat Tonbul, Nefise Dilek, Tuncer Hökelek

**Affiliations:** a Department of Physics, Hacettepe University, 06800 Beytepe, Ankara, Turkey; b Department of Chemistry, Kafkas University, 36100 Kars, Turkey; c Aksaray University, Department of Physics, 68100, Aksaray, Turkey

**Keywords:** crystal structure, zinc(II) complex, benzoic acid, nicotinamide derivatives, hydrogen bonding

## Abstract

The asymmetric unit of the title coordination polymer, [Zn(C_7_H_4_NO_4_)_2_(C_6_H_6_N_2_O)_2_]_*n*_, contains two 4-nitro­benzoate (NB) anions and two nicotinamide (NA) ligands. The Zn^II^ atom has a slightly distorted octa­hedral coordination sphere. In the equatorial plane, it is coordinated by three carboxyl­ate O atoms of the NB anions and one O atom of one of the two NA ligands. The axial positions are occupied by the pyridine N atoms of the two NA ligands. In the two NB anions, the carboxyl­ate groups are twisted away from the attached benzene rings by 13.8 (2) and 13.4 (2)°, while the benzene rings are oriented at a dihedral angle of 11.5 (2)°. The dihedral angle between the NA rings is 10.3 (1)°. Only one of the two NB anions and one of the two NA ligands bridge adjacent Zn^II^ ions through eight- and twelve-membered rings, respectively, forming polymeric chains running along the *a*-axis direction. In the crystal, N—H ⋯ O hydrogen bonds link adjacent chains, enclosing *R*(16), *R*_2_^2^(20) and *R*_6_^6^(16) ring motifs, forming layers parallel to (01-1). The layers are linked *via* a number of C—H⋯O hydrogen bonds, forming a three-dimensional network.

## Abstract 2

The asymmetric unit of the title zinc(II) coordination polymer contains two 4-nitro­benzoate (NB) anions and two nicotinamide (NA) ligands. Only one of the two NB anions and one of the two NA ligands bridge adjacent Zn^II^ ions through eight- and 12-membered rings, respectively, forming polymeric chains running along the *a*-axis direction.

## Chemical context   

Nicotinamide (NA) is one form of niacin. A deficiency of this vitamin leads to loss of copper from the body, known as pellagra disease. Victims of pellagra show unusually high serum and urinary copper levels (Krishnamachari, 1974 ▸). The nicotinic acid derivative *N*,*N*-di­ethyl­nicotinamide (DENA) is an important respiratory stimulant (Bigoli *et al.*, 1972 ▸). Trans­ition metal complexes with biochemical mol­ecules show inter­esting physical and/or chemical properties, through which they may find applications in biological systems (Antolini *et al.*, 1982 ▸). Some benzoic acid derivatives, such as 4-amino­benzoic acid, have been extensively reported in coordination chemistry, as bifunctional organic ligands, due to the varieties of their coordination modes (Chen & Chen, 2002 ▸; Amiraslanov *et al.*, 1979 ▸; Hauptmann *et al.*, 2000 ▸).

The structure–function–coordination relationships of the aryl­carboxyl­ate ion in Zn^II^ complexes of benzoic acid deriv­atives change depending on the nature and position of the substituent groups on the benzene ring, the nature of the additional ligand mol­ecule or solvent, and the pH and temperature of synthesis (Shnulin *et al.*, 1981 ▸; Nadzhafov *et al.*, 1981 ▸; Antsyshkina *et al.*, 1980 ▸; Adiwidjaja *et al.*, 1978 ▸). When pyridine and its derivatives are used instead of water mol­ecules, the structure is completely different (Catterick *et al.*, 1974 ▸).
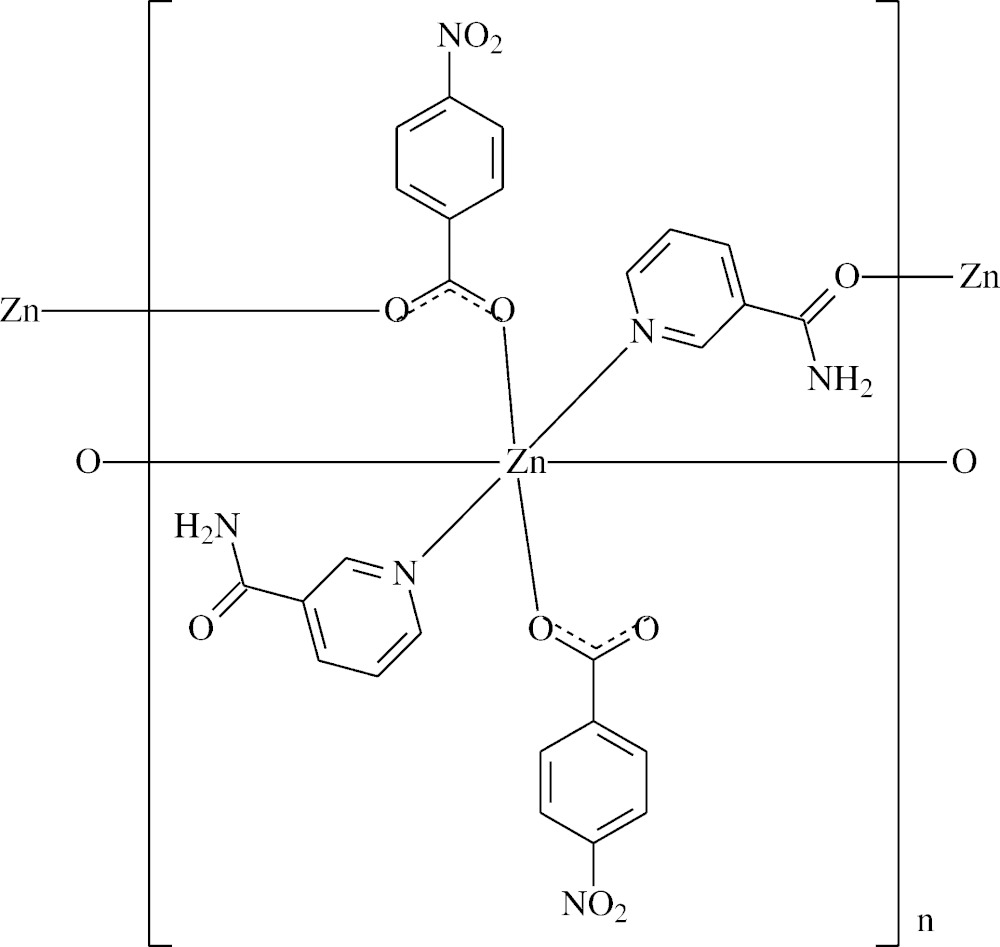


## Structural commentary   

**Figure 1:**
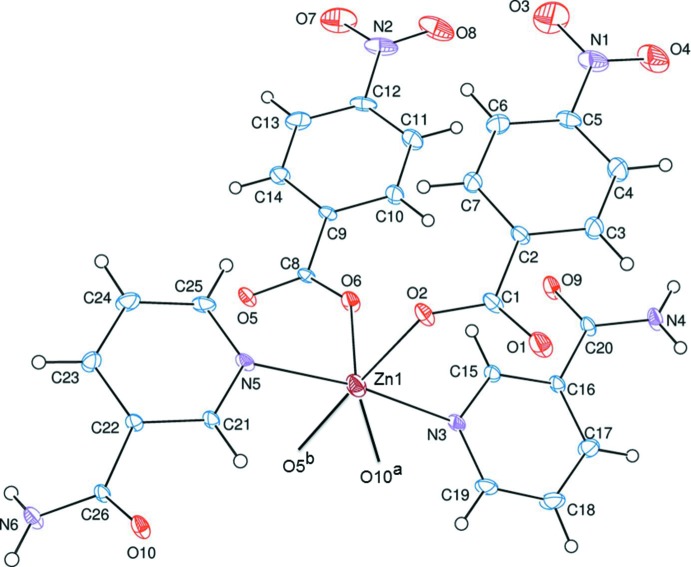
The asymmetric unit of the title mol­ecule, with the atom labelling. Displacement ellipsoids are drawn at the 50% probability level [symmetry codes: (a) −*x* + 1, −*y*, −*z*; (b) −*x* + 2, −*y*, −*z*].

**Figure 2:**
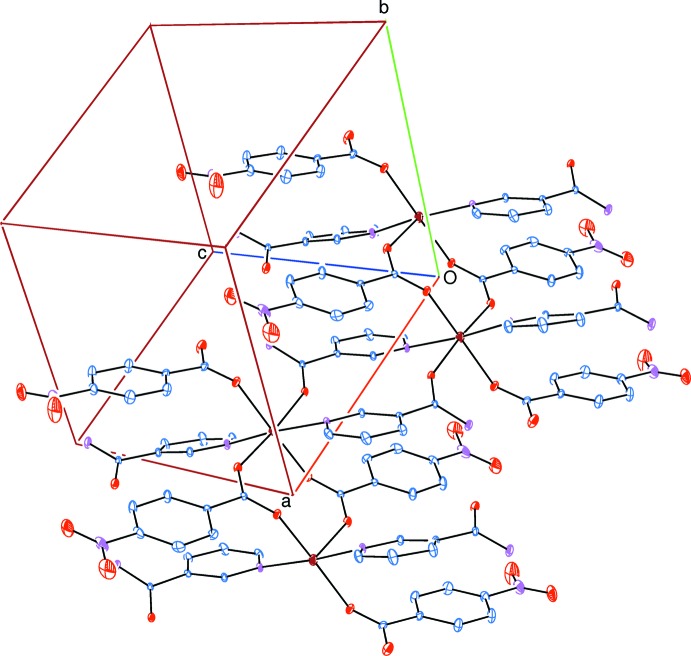
A partial view of the crystal packing of the title compound. H atoms have been omitted for clarity.

**Figure 3:**
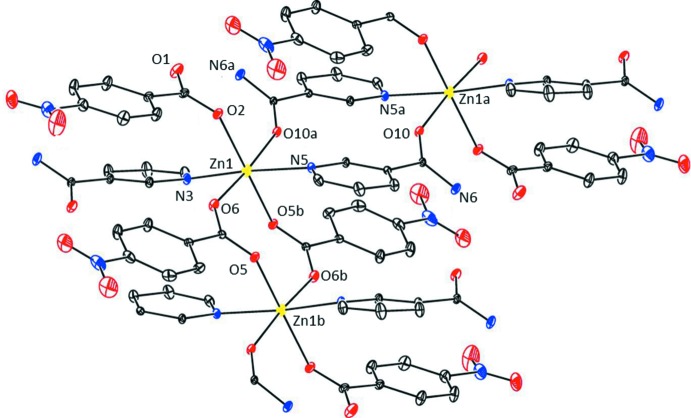
Part of the crystal packing of the title compound, showing the eight- and twelve-membered rings [symmetry codes (a) −*x* + 1, −*y*, −*z*; (b) −*x* + 2, −*y*, −*z*]. H atoms have been omitted for clarity.

The asymmetric unit of the title polymeric compound contains two 4-nitro­benzoate (NB) anions and two nicotinamide (NA) ligands; the NB anions act as monodentate ligands (Fig. 1 ▸). Only one of the two NB anions and one of the two NA ligands bridge the adjacent Zn^II^ ions through eight- and twelve-membered rings, respectively, forming polymeric chains running along the *a*-axis direction (Fig. 2 ▸). In the eight- and twelve-membered rings, the distances between the symmetry related ions [Zn1⋯Zn1^a^, N5⋯N5^a^, O10⋯O10^a^, and Zn1⋯Zn1^b^, O5⋯O5^b^, O6⋯O6^b^ are 7.3237 (6), 5.855 (4), 4.480 (3) Å and 4.67 (6), 3.668 (4), 4.256 (4) Å, respectively [symmetry codes (a) = −*x* + 1, − *y*, −*z*; (b) − *x* + 2, −*y*, − *z*]; see Fig. 3 ▸.

The three carboxyl­ate O atoms (O2, O5 and O6) of the three NB anions and one O atom (O10) of one of the two NA ligands in the equatorial plane around the Zn^II^ cation form a slightly distorted square-planar arrangement, while the slightly distorted octa­hedral coordination is completed by the two pyridine N atoms (N3 and N5) of the two NA ligands in the axial positions (Table 1 ▸ and Fig. 3 ▸).

The near equality of the C1—O1 [1.247 (4) Å], C1—O2 [1.261 (4) Å], C8—O5 [1.248 (4) Å] and C8—O6 [1.255 (4) Å] bonds in the carboxyl­ate groups indicate delocalized bonding arrangements, rather than localized single and double bonds. The average Zn—O_carboxyl­ate_ and Zn—N distances are 2.147 (2) Å and 2.285 (3) Å, respectively, while Zn1—O10 distance is 2.280 (2) Å. The Zn1 atom lies 1.4330 (4) Å and 0.1897 (4) Å above the planar (O1/O2/C1) and (O5/O6/C8) carboxyl­ate groups, respectively. The average O—Zn—O and O—Zn—N bond angles are 89.93 (10) and 89.99 (10)°, respectively.

The dihedral angles between the planar carboxyl­ate groups [(O1/O2/C1) and (O5/O6/C8)] and the adjacent benzene rings [*A* (C2—C7) and *B* (C9—C14)] are 13.8 (2) and 13.4 (2)°, respectively, while the benzene rings are oriented at a dihedral angle of 11.5 (2)°. The dihedral angle between the nicotin­amide rings [*C* (N3/C15—C19) and *D* (N5/C21—C25)] is 10.3 (1)°, and they are oriented with respect to benzene rings *A* and *B* at dihedral angles of *A*/*C* = 17.3 (1), *A*/D = 7.7 (1), *B*/*C* = 28.8 (1) and *B*/*D* = 18.9 (1)°.

## Supra­molecular features   

**Figure 4:**
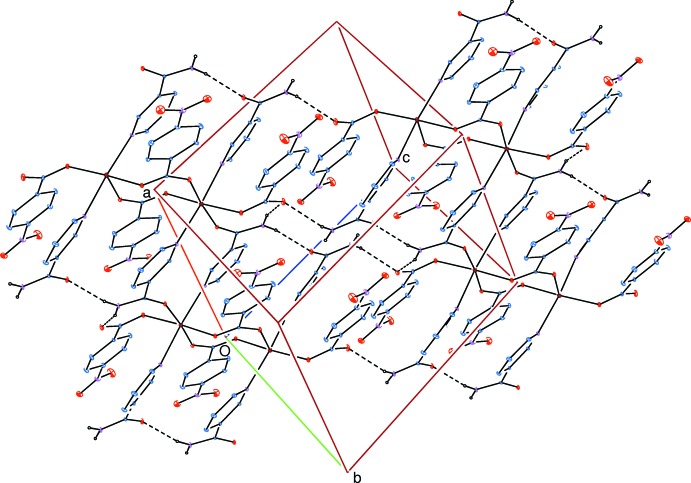
Part of the crystal packing of the title compound with the N—H⋯O hydrogen bonds shown as dashed lines (see Table 1 ▸ for details; other H atoms have been omitted for clarity).

In the crystal, strong N—H⋯O_c_ (c = carboxylate) and N—H⋯O_n_ (n = nicotinamide) hydrogen bonds (Table 2 ▸) link adjacent chains through *R*(16), 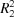
(20) and 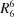
(16) ring motifs (Bernstein *et al.*, 1995 ▸) into layers parallel to (01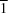) (Fig. 4 ▸). Weak intra­molecular C—H_n_⋯O_c_ and inter­mol­ec­ular C—H_n_⋯O_nb_ (nb = nitro­benzoate) and C—H_n_⋯O_n_ hydrogen bonds (Table 1 ▸) link the layers into a three-dimensional framework.

## Synthesis and crystallization   

The title compound was prepared by the reaction of ZnSO_4_·H_2_O (0.89 g, 5 mmol) in H_2_O (25 ml) and nicotinamide (1.22 g, 10 mmol) in H_2_O (25 ml) with sodium 4-nitro­benzoate (1.90 g, 10 mmol) in H_2_O (150 ml). The mixture was filtered and set aside to crystallize at ambient temperature for one week, giving yellow block-like crystals.

## Refinement   

The experimental details including the crystal data, data collection and refinement are summarized in Table 3 ▸. H atoms were positioned geometrically and constrained to ride on their parent atoms, with C—H = 0.93 Å and N—H = 0.86 Å, and with *U*_iso_(H) = 1.2*U*_eq_(C,N). The highest residual electron density and the deepest hole were found 0.29 Å and 0.48 Å from atoms N6 and Zn1, respectively.

## Supplementary Material

Crystal structure: contains datablock(s) I, global. DOI: 10.1107/S2056989015006490/su5099sup1.cif

Structure factors: contains datablock(s) I. DOI: 10.1107/S2056989015006490/su5099Isup2.hkl

CCDC reference: 1057121

Additional supporting information:  crystallographic information; 3D view; checkCIF report

## Figures and Tables

**Table 1:** Selected bond lengths ()

Zn1O2	2.140(2)	Zn1O10^ii^	2.280(2)
Zn1O5^i^	2.142(2)	Zn1N3	2.288(3)
Zn1O6	2.160(2)	Zn1N5	2.282(3)

Symmetry codes: (i) 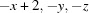; (ii) 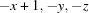.

**Table 2:** Hydrogen-bond geometry (, )

*D*H*A*	*D*H	H*A*	*D* *A*	*D*H*A*
N4H4*B*O1^i^	0.86	2.20	2.851(4)	132
N6H6*A*O1^ii^	0.86	1.98	2.814(4)	164
N6H6*B*O9^iii^	0.86	2.09	2.880(4)	153
C15H15O6	0.93	2.45	3.079(4)	125
C19H19O3^iv^	0.93	2.57	3.238(7)	130
C21H21O10^ii^	0.93	2.38	3.058(4)	130

Symmetry codes: (i) 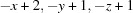; (ii) 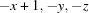; (iii) 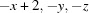; (iv) 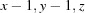.

**Table 3:** Experimental details

Crystal data	
Chemical formula	[Zn(C_6_H_6_N_2_O)_2_(C_7_H_4_NO_4_)_2_]
*M*_r_	641.87
Crystal system, space group	Triclinic, *P* 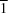
Temperature (K)	296
*a*, *b*, *c* ()	9.5118(3), 10.5591(3), 14.5326(5)
, , ()	109.846(4), 93.618(3), 104.815(4)
*V* (^3^)	1309.11(9)
*Z*	2
Radiation type	Mo *K*
(mm^1^)	1.01
Crystal size (mm)	0.50 0.37 0.33

Data collection	
Diffractometer	Bruker SMART BREEZE CCD
Absorption correction	Multi-scan (*SADABS*; Bruker, 2012 ▸)
*T*_min_, *T*_max_	0.635, 0.705
No. of measured, independent and observed [*I* > 2(*I*)] reflections	34333, 6520, 5816
*R*_int_	0.023
(sin /)_max_ (^1^)	0.669

Refinement	
*R*[*F* ^2^ > 2(*F* ^2^)], *wR*(*F* ^2^), *S*	0.073, 0.238, 1.09
No. of reflections	6520
No. of parameters	388
H-atom treatment	H-atom parameters constrained
_max_, _min_ (e ^3^)	1.29, 0.60

Computer programs: *APEX2* and *SAINT* (Bruker, 2012 ▸), *SHELXS97* and *SHELXL97* (Sheldrick, 2008 ▸), *ORTEP-3* for Windows and *WinGX* (Farrugia, 2012 ▸) and *PLATON* (Spek, 2009 ▸).

## supplementary crystallographic information

### Crystal data

**Table:**

[Zn(C_6_H_6_N_2_O)_2_(C_7_H_4_NO_4_)_2_]	*Z* = 2
*M**_r_* = 641.87	*F*(000) = 656
Triclinic, *P*1	*D*_x_ = 1.628 Mg m^−^^3^
Hall symbol: -P 1	Mo *K*α radiation, λ = 0.71073 Å
*a* = 9.5118 (3) Å	Cell parameters from 9922 reflections
*b* = 10.5591 (3) Å	θ = 2.5–28.4°
*c* = 14.5326 (5) Å	µ = 1.01 mm^−^^1^
α = 109.846 (4)°	*T* = 296 K
β = 93.618 (3)°	Block, yellow
γ = 104.815 (4)°	0.50 × 0.37 × 0.33 mm
*V* = 1309.11 (9) Å^3^	

### Data collection

**Table:**

Bruker SMART BREEZE CCD diffractometer	6520 independent reflections
Radiation source: fine-focus sealed tube	5816 reflections with *I* > 2σ(*I*)
Graphite monochromator	*R*_int_ = 0.023
φ and ω scans	θ_max_ = 28.4°, θ_min_ = 1.5°
Absorption correction: multi-scan (*SADABS*; Bruker, 2012)	*h* = −12→12
*T*_min_ = 0.635, *T*_max_ = 0.705	*k* = −13→14
34333 measured reflections	*l* = −19→19

### Refinement

**Table:**

Refinement on *F*^2^	Primary atom site location: structure-invariant direct methods
Least-squares matrix: full	Secondary atom site location: difference Fourier map
*R*[*F*^2^ > 2σ(*F*^2^)] = 0.073	Hydrogen site location: inferred from neighbouring sites
*wR*(*F*^2^) = 0.238	H-atom parameters constrained
*S* = 1.09	*w* = 1/[σ^2^(*F*_o_^2^) + (0.149*P*)^2^ + 3.1586*P*] where *P* = (*F*_o_^2^ + 2*F*_c_^2^)/3
6520 reflections	(Δ/σ)_max_ < 0.001
388 parameters	Δρ_max_ = 1.29 e Å^−^^3^
0 restraints	Δρ_min_ = −0.59 e Å^−^^3^

### Special details

**Table:**

Geometry. All esds (except the esd in the dihedral angle between two l.s. planes) are estimated using the full covariance matrix. The cell esds are taken into account individually in the estimation of esds in distances, angles and torsion angles; correlations between esds in cell parameters are only used when they are defined by crystal symmetry. An approximate (isotropic) treatment of cell esds is used for estimating esds involving l.s. planes.
Refinement. Refinement of F^2^ against ALL reflections. The weighted R-factor wR and goodness of fit S are based on F^2^, conventional R-factors R are based on F, with F set to zero for negative F^2^. The threshold expression of F^2^ > 2sigma(F^2^) is used only for calculating R-factors(gt) etc. and is not relevant to the choice of reflections for refinement. R-factors based on F^2^ are statistically about twice as large as those based on F, and R- factors based on ALL data will be even larger.

### Fractional atomic coordinates and isotropic or equivalent isotropic displacement parameters (Å^2^)

**Table:**

	*x*	*y*	*z*	*U*_iso_*/*U*_eq_	
Zn1	0.88909 (4)	0.14132 (4)	0.11465 (3)	0.02880 (19)	
O1	0.8671 (3)	0.4391 (3)	0.3500 (2)	0.0410 (8)	
O2	0.9117 (3)	0.3588 (3)	0.19518 (19)	0.0266 (5)	
O3	1.5920 (5)	0.8555 (6)	0.3452 (4)	0.0923 (19)	
O4	1.5777 (4)	0.8861 (4)	0.4965 (3)	0.0560 (10)	
O5	1.1523 (3)	0.0826 (2)	−0.05180 (18)	0.0230 (5)	
O6	1.1170 (3)	0.2086 (3)	0.09758 (19)	0.0266 (5)	
O7	1.8536 (4)	0.5337 (5)	0.0411 (4)	0.0741 (14)	
O8	1.8302 (4)	0.6372 (4)	0.1922 (4)	0.0651 (12)	
O9	1.4003 (3)	0.3463 (3)	0.3975 (2)	0.0315 (6)	
O10	0.3467 (2)	−0.0828 (3)	−0.14454 (17)	0.0235 (5)	
N1	1.5271 (4)	0.8314 (4)	0.4092 (3)	0.0437 (9)	
N2	1.7850 (4)	0.5475 (4)	0.1102 (4)	0.0464 (10)	
N3	0.9685 (3)	0.1292 (3)	0.2625 (2)	0.0209 (5)	
N4	1.3297 (3)	0.4235 (4)	0.5466 (2)	0.0301 (7)	
H4A	1.4152	0.4830	0.5733	0.036*	
H4B	1.2605	0.4171	0.5816	0.036*	
N5	0.7923 (3)	0.1216 (3)	−0.0391 (2)	0.0192 (5)	
N6	0.3979 (3)	−0.2171 (3)	−0.2888 (2)	0.0257 (6)	
H6A	0.3090	−0.2722	−0.3076	0.031*	
H6B	0.4633	−0.2315	−0.3263	0.031*	
C1	0.9464 (4)	0.4401 (3)	0.2850 (3)	0.0231 (6)	
C2	1.0981 (4)	0.5454 (3)	0.3168 (2)	0.0212 (6)	
C3	1.1568 (4)	0.6153 (4)	0.4178 (3)	0.0337 (8)	
H3	1.1008	0.5981	0.4648	0.040*	
C4	1.2969 (5)	0.7097 (5)	0.4486 (3)	0.0377 (9)	
H4	1.3356	0.7566	0.5157	0.045*	
C5	1.3777 (4)	0.7324 (4)	0.3772 (3)	0.0295 (8)	
C6	1.3225 (4)	0.6662 (5)	0.2774 (3)	0.0351 (8)	
H6	1.3786	0.6851	0.2309	0.042*	
C7	1.1821 (4)	0.5709 (4)	0.2470 (3)	0.0298 (8)	
H7	1.1444	0.5241	0.1798	0.036*	
C8	1.1944 (3)	0.1804 (3)	0.0309 (2)	0.0161 (5)	
C9	1.3506 (3)	0.2755 (3)	0.0529 (2)	0.0170 (6)	
C10	1.4151 (4)	0.3680 (4)	0.1489 (3)	0.0291 (7)	
H10	1.3613	0.3707	0.2004	0.035*	
C11	1.5580 (4)	0.4561 (5)	0.1691 (3)	0.0359 (9)	
H11	1.6008	0.5176	0.2333	0.043*	
C12	1.6357 (4)	0.4502 (4)	0.0910 (3)	0.0300 (8)	
C13	1.5764 (4)	0.3579 (4)	−0.0052 (3)	0.0334 (8)	
H13	1.6314	0.3547	−0.0562	0.040*	
C14	1.4334 (4)	0.2706 (4)	−0.0237 (3)	0.0269 (7)	
H14	1.3919	0.2079	−0.0879	0.032*	
C15	1.1025 (3)	0.2154 (3)	0.3102 (2)	0.0198 (6)	
H15	1.1637	0.2597	0.2758	0.024*	
C16	1.1542 (3)	0.2416 (3)	0.4080 (2)	0.0188 (6)	
C17	1.0644 (4)	0.1754 (4)	0.4601 (3)	0.0308 (8)	
H17	1.0946	0.1935	0.5266	0.037*	
C18	0.9282 (4)	0.0815 (5)	0.4100 (3)	0.0363 (9)	
H18	0.8671	0.0319	0.4417	0.044*	
C19	0.8841 (4)	0.0623 (4)	0.3123 (3)	0.0275 (7)	
H19	0.7920	0.0002	0.2799	0.033*	
C20	1.3046 (3)	0.3414 (3)	0.4512 (2)	0.0203 (6)	
C21	0.6540 (3)	0.0355 (3)	−0.0750 (2)	0.0176 (6)	
H21	0.5976	0.0097	−0.0307	0.021*	
C22	0.5908 (3)	−0.0170 (3)	−0.1739 (2)	0.0154 (5)	
C23	0.6756 (4)	0.0196 (4)	−0.2406 (3)	0.0247 (7)	
H23	0.6386	−0.0170	−0.3081	0.030*	
C24	0.8167 (4)	0.1120 (4)	−0.2041 (3)	0.0299 (8)	
H24	0.8746	0.1411	−0.2466	0.036*	
C25	0.8706 (3)	0.1609 (4)	−0.1032 (3)	0.0241 (7)	
H25	0.9651	0.2234	−0.0794	0.029*	
C26	0.4345 (3)	−0.1097 (3)	−0.2019 (2)	0.0156 (5)	

### Atomic displacement parameters (Å^2^)

**Table:**

	*U*^11^	*U*^22^	*U*^33^	*U*^12^	*U*^13^	*U*^23^
Zn1	0.0193 (3)	0.0303 (3)	0.0256 (3)	0.00170 (18)	0.00168 (17)	0.00104 (19)
O1	0.0224 (13)	0.0400 (16)	0.0361 (16)	−0.0070 (11)	0.0135 (11)	−0.0058 (12)
O2	0.0249 (12)	0.0188 (11)	0.0245 (12)	0.0026 (9)	−0.0005 (9)	−0.0028 (9)
O3	0.045 (2)	0.110 (4)	0.081 (3)	−0.040 (3)	0.011 (2)	0.031 (3)
O4	0.0332 (17)	0.050 (2)	0.062 (2)	−0.0093 (15)	−0.0172 (16)	0.0128 (18)
O5	0.0187 (11)	0.0177 (11)	0.0223 (11)	0.0009 (9)	−0.0003 (9)	−0.0014 (9)
O6	0.0118 (11)	0.0315 (13)	0.0234 (12)	−0.0019 (9)	0.0054 (9)	−0.0002 (10)
O7	0.0295 (18)	0.081 (3)	0.101 (4)	−0.0137 (18)	0.022 (2)	0.040 (3)
O8	0.0286 (17)	0.046 (2)	0.096 (3)	−0.0137 (15)	−0.0084 (19)	0.018 (2)
O9	0.0135 (11)	0.0370 (14)	0.0249 (12)	−0.0004 (10)	0.0065 (9)	−0.0068 (10)
O10	0.0099 (10)	0.0289 (12)	0.0188 (11)	0.0011 (9)	0.0031 (8)	−0.0038 (9)
N1	0.0233 (16)	0.0369 (19)	0.059 (2)	−0.0085 (14)	−0.0031 (16)	0.0177 (18)
N2	0.0146 (15)	0.040 (2)	0.085 (3)	−0.0043 (14)	0.0024 (17)	0.033 (2)
N3	0.0121 (12)	0.0215 (13)	0.0204 (13)	−0.0026 (10)	−0.0018 (9)	0.0036 (10)
N4	0.0171 (13)	0.0356 (17)	0.0186 (14)	−0.0027 (12)	0.0024 (10)	−0.0055 (12)
N5	0.0084 (11)	0.0199 (12)	0.0218 (13)	−0.0016 (9)	−0.0011 (9)	0.0034 (10)
N6	0.0143 (12)	0.0277 (15)	0.0193 (13)	−0.0015 (10)	0.0047 (10)	−0.0054 (11)
C1	0.0163 (14)	0.0167 (14)	0.0266 (16)	0.0015 (11)	0.0037 (12)	−0.0015 (12)
C2	0.0168 (14)	0.0168 (14)	0.0223 (15)	0.0006 (11)	0.0027 (11)	0.0012 (11)
C3	0.0270 (18)	0.038 (2)	0.0215 (16)	−0.0058 (15)	0.0042 (13)	0.0036 (15)
C4	0.030 (2)	0.041 (2)	0.0248 (18)	−0.0070 (17)	−0.0043 (15)	0.0061 (16)
C5	0.0173 (15)	0.0241 (17)	0.039 (2)	−0.0046 (13)	−0.0007 (14)	0.0107 (15)
C6	0.0282 (19)	0.037 (2)	0.034 (2)	−0.0034 (15)	0.0099 (15)	0.0140 (16)
C7	0.0275 (18)	0.0305 (18)	0.0213 (16)	−0.0030 (14)	0.0043 (13)	0.0055 (14)
C8	0.0091 (12)	0.0141 (13)	0.0211 (14)	0.0007 (10)	0.0018 (10)	0.0037 (11)
C9	0.0098 (12)	0.0141 (13)	0.0237 (15)	0.0011 (10)	0.0036 (11)	0.0044 (11)
C10	0.0178 (15)	0.0339 (19)	0.0231 (16)	−0.0056 (13)	0.0014 (12)	0.0052 (14)
C11	0.0220 (17)	0.037 (2)	0.0325 (19)	−0.0087 (15)	−0.0044 (14)	0.0066 (16)
C12	0.0115 (14)	0.0246 (17)	0.053 (2)	−0.0024 (12)	0.0013 (14)	0.0205 (16)
C13	0.0211 (17)	0.036 (2)	0.047 (2)	0.0062 (15)	0.0187 (16)	0.0191 (18)
C14	0.0214 (16)	0.0251 (16)	0.0289 (17)	0.0023 (13)	0.0110 (13)	0.0056 (13)
C15	0.0125 (13)	0.0224 (15)	0.0176 (14)	−0.0010 (11)	0.0008 (10)	0.0035 (11)
C16	0.0115 (13)	0.0202 (14)	0.0176 (14)	0.0003 (11)	−0.0003 (10)	0.0020 (11)
C17	0.0218 (16)	0.041 (2)	0.0258 (17)	−0.0033 (15)	−0.0018 (13)	0.0171 (16)
C18	0.0227 (17)	0.045 (2)	0.037 (2)	−0.0087 (16)	−0.0014 (15)	0.0245 (18)
C19	0.0145 (14)	0.0291 (17)	0.0321 (18)	−0.0062 (12)	−0.0023 (12)	0.0133 (14)
C20	0.0119 (13)	0.0220 (15)	0.0178 (14)	0.0015 (11)	−0.0009 (10)	−0.0009 (12)
C21	0.0087 (12)	0.0214 (14)	0.0163 (13)	−0.0003 (10)	0.0006 (10)	0.0028 (11)
C22	0.0083 (12)	0.0182 (13)	0.0172 (13)	0.0027 (10)	0.0024 (10)	0.0041 (11)
C23	0.0169 (15)	0.0356 (18)	0.0205 (15)	0.0023 (13)	0.0042 (12)	0.0129 (14)
C24	0.0168 (15)	0.041 (2)	0.0340 (19)	−0.0005 (14)	0.0056 (13)	0.0225 (16)
C25	0.0106 (13)	0.0252 (16)	0.0347 (18)	−0.0020 (11)	0.0005 (12)	0.0150 (14)
C26	0.0085 (12)	0.0188 (13)	0.0142 (12)	0.0013 (10)	0.0008 (9)	0.0019 (11)

### Geometric parameters (Å, º)

**Table:**

Zn1—O2	2.140 (2)	C5—C6	1.377 (6)
Zn1—O5^i^	2.142 (2)	C6—H6	0.9300
Zn1—O6	2.160 (2)	C7—C6	1.389 (5)
Zn1—O10^ii^	2.280 (2)	C7—H7	0.9300
Zn1—N3	2.288 (3)	C8—C9	1.509 (4)
Zn1—N5	2.282 (3)	C9—C10	1.392 (5)
O1—C1	1.247 (4)	C9—C14	1.399 (4)
O2—C1	1.261 (4)	C10—C11	1.385 (5)
O4—N1	1.208 (6)	C10—H10	0.9300
O5—Zn1^i^	2.142 (2)	C11—H11	0.9300
O5—C8	1.248 (4)	C12—C11	1.384 (6)
O6—C8	1.255 (4)	C13—C12	1.384 (6)
O9—C20	1.239 (4)	C13—C14	1.384 (5)
O10—Zn1^ii^	2.280 (2)	C13—H13	0.9300
N1—O3	1.210 (6)	C14—H14	0.9300
N2—O7	1.220 (7)	C15—C16	1.383 (4)
N2—O8	1.210 (7)	C15—H15	0.9300
N2—C12	1.469 (4)	C16—C17	1.388 (5)
N3—C15	1.342 (4)	C16—C20	1.489 (4)
N3—C19	1.341 (4)	C17—H17	0.9300
N4—H4A	0.8600	C18—C17	1.388 (5)
N4—H4B	0.8600	C18—H18	0.9300
N5—C21	1.345 (4)	C19—C18	1.384 (5)
N5—C25	1.337 (4)	C19—H19	0.9300
N6—C26	1.332 (4)	C20—N4	1.329 (4)
N6—H6A	0.8600	C21—H21	0.9300
N6—H6B	0.8600	C22—C21	1.384 (4)
C1—C2	1.510 (4)	C22—C23	1.391 (4)
C2—C3	1.401 (5)	C23—C24	1.387 (5)
C2—C7	1.384 (5)	C23—H23	0.9300
C3—C4	1.383 (5)	C24—H24	0.9300
C3—H3	0.9300	C25—C24	1.391 (5)
C4—H4	0.9300	C25—H25	0.9300
C5—N1	1.468 (5)	C26—O10	1.233 (4)
C5—C4	1.379 (6)	C26—C22	1.497 (4)
			
O2—Zn1—O5^i^	170.95 (10)	O5—C8—O6	125.6 (3)
O2—Zn1—O6	86.90 (10)	O5—C8—C9	117.8 (3)
O2—Zn1—O10^ii^	89.70 (10)	O6—C8—C9	116.6 (3)
O2—Zn1—N3	87.21 (10)	C10—C9—C8	121.1 (3)
O2—Zn1—N5	99.61 (10)	C10—C9—C14	119.0 (3)
O5^i^—Zn1—O6	99.77 (10)	C14—C9—C8	119.9 (3)
O5^i^—Zn1—O10^ii^	83.34 (9)	C9—C10—H10	119.4
O5^i^—Zn1—N3	86.87 (10)	C11—C10—C9	121.1 (3)
O5^i^—Zn1—N5	85.89 (10)	C11—C10—H10	119.4
O6—Zn1—O10^ii^	175.66 (8)	C10—C11—H11	120.9
O6—Zn1—N3	88.43 (10)	C12—C11—C10	118.3 (4)
O6—Zn1—N5	95.75 (10)	C12—C11—H11	120.9
O10^ii^—Zn1—N3	88.71 (9)	C11—C12—N2	119.1 (4)
O10^ii^—Zn1—N5	87.48 (9)	C13—C12—N2	118.6 (4)
N5—Zn1—N3	172.16 (10)	C13—C12—C11	122.3 (3)
C1—O2—Zn1	135.7 (2)	C12—C13—H13	120.7
C8—O5—Zn1^i^	138.9 (2)	C14—C13—C12	118.5 (3)
C8—O6—Zn1	137.8 (2)	C14—C13—H13	120.7
C26—O10—Zn1^ii^	147.3 (2)	C9—C14—H14	119.6
O3—N1—C5	117.1 (4)	C13—C14—C9	120.8 (3)
O4—N1—O3	123.5 (4)	C13—C14—H14	119.6
O4—N1—C5	119.3 (4)	N3—C15—C16	123.3 (3)
O7—N2—C12	117.3 (4)	N3—C15—H15	118.3
O8—N2—O7	124.5 (4)	C16—C15—H15	118.3
O8—N2—C12	118.2 (4)	C15—C16—C17	119.0 (3)
C15—N3—Zn1	116.4 (2)	C15—C16—C20	117.1 (3)
C19—N3—Zn1	125.3 (2)	C17—C16—C20	123.9 (3)
C19—N3—C15	117.4 (3)	C16—C17—H17	121.0
C20—N4—H4A	120.0	C18—C17—C16	118.0 (3)
C20—N4—H4B	120.0	C18—C17—H17	121.0
H4A—N4—H4B	120.0	C17—C18—H18	120.3
C21—N5—Zn1	115.9 (2)	C19—C18—C17	119.3 (3)
C25—N5—Zn1	124.9 (2)	C19—C18—H18	120.3
C25—N5—C21	117.3 (3)	N3—C19—C18	122.9 (3)
C26—N6—H6A	120.0	N3—C19—H19	118.5
C26—N6—H6B	120.0	C18—C19—H19	118.5
H6A—N6—H6B	120.0	O9—C20—N4	122.6 (3)
O1—C1—O2	125.5 (3)	O9—C20—C16	119.4 (3)
O1—C1—C2	117.3 (3)	N4—C20—C16	118.0 (3)
O2—C1—C2	117.1 (3)	N5—C21—C22	123.9 (3)
C3—C2—C1	119.9 (3)	N5—C21—H21	118.0
C7—C2—C1	120.6 (3)	C22—C21—H21	118.0
C7—C2—C3	119.4 (3)	C21—C22—C23	118.2 (3)
C2—C3—H3	119.5	C21—C22—C26	117.5 (3)
C4—C3—C2	120.9 (3)	C23—C22—C26	124.3 (3)
C4—C3—H3	119.5	C22—C23—H23	120.8
C3—C4—H4	120.9	C24—C23—C22	118.4 (3)
C5—C4—C3	118.2 (4)	C24—C23—H23	120.8
C5—C4—H4	120.9	C23—C24—C25	119.4 (3)
C6—C5—C4	122.2 (3)	C23—C24—H24	120.3
C6—C5—N1	119.2 (4)	C25—C24—H24	120.3
C4—C5—N1	118.7 (4)	N5—C25—C24	122.7 (3)
C5—C6—C7	119.3 (3)	N5—C25—H25	118.7
C5—C6—H6	120.4	C24—C25—H25	118.7
C7—C6—H6	120.4	O10—C26—N6	123.5 (3)
C2—C7—C6	120.0 (3)	O10—C26—C22	119.1 (3)
C2—C7—H7	120.0	N6—C26—C22	117.4 (3)
C6—C7—H7	120.0		
			
O6—Zn1—O2—C1	−91.7 (3)	C1—C2—C7—C6	−178.9 (4)
O10^ii^—Zn1—O2—C1	85.6 (3)	C3—C2—C7—C6	−0.7 (6)
N3—Zn1—O2—C1	−3.1 (3)	C2—C3—C4—C5	−0.4 (7)
N5—Zn1—O2—C1	173.0 (3)	C4—C5—N1—O3	−175.7 (5)
O2—Zn1—O6—C8	−138.6 (4)	C4—C5—N1—O4	2.9 (6)
O5^i^—Zn1—O6—C8	47.6 (4)	C6—C5—N1—O3	3.8 (7)
N3—Zn1—O6—C8	134.1 (4)	C6—C5—N1—O4	−177.6 (4)
N5—Zn1—O6—C8	−39.2 (4)	N1—C5—C4—C3	−179.4 (4)
O2—Zn1—N3—C15	−59.4 (2)	C6—C5—C4—C3	1.0 (7)
O2—Zn1—N3—C19	108.8 (3)	N1—C5—C6—C7	179.0 (4)
O5^i^—Zn1—N3—C15	127.5 (2)	C4—C5—C6—C7	−1.5 (7)
O5^i^—Zn1—N3—C19	−64.4 (3)	C2—C7—C6—C5	1.3 (6)
O6—Zn1—N3—C15	27.6 (2)	O5—C8—C9—C10	−167.0 (3)
O6—Zn1—N3—C19	−164.2 (3)	O5—C8—C9—C14	12.4 (4)
O10^ii^—Zn1—N3—C15	−149.2 (2)	O6—C8—C9—C10	14.2 (5)
O10^ii^—Zn1—N3—C19	19.0 (3)	O6—C8—C9—C14	−166.4 (3)
O2—Zn1—N5—C21	−115.0 (2)	C8—C9—C10—C11	−179.5 (4)
O2—Zn1—N5—C25	81.2 (3)	C14—C9—C10—C11	1.1 (6)
O5^i^—Zn1—N5—C21	57.8 (2)	C8—C9—C14—C13	179.4 (3)
O5^i^—Zn1—N5—C25	−106.0 (3)	C10—C9—C14—C13	−1.2 (5)
O6—Zn1—N5—C21	157.2 (2)	C9—C10—C11—C12	0.1 (6)
O6—Zn1—N5—C25	−6.6 (3)	N2—C12—C11—C10	177.2 (4)
O10^ii^—Zn1—N5—C21	−25.7 (2)	C13—C12—C11—C10	−1.3 (6)
O10^ii^—Zn1—N5—C25	170.5 (3)	C14—C13—C12—N2	−177.2 (4)
Zn1—O2—C1—O1	−73.4 (5)	C14—C13—C12—C11	1.2 (6)
Zn1—O2—C1—C2	105.2 (4)	C12—C13—C14—C9	0.1 (6)
Zn1^i^—O5—C8—O6	−77.3 (4)	N3—C15—C16—C17	0.4 (5)
Zn1^i^—O5—C8—C9	104.0 (3)	N3—C15—C16—C20	−179.1 (3)
Zn1—O6—C8—O5	−7.5 (6)	C15—C16—C17—C18	2.4 (6)
Zn1—O6—C8—C9	171.2 (2)	C20—C16—C17—C18	−178.1 (4)
O7—N2—C12—C11	175.7 (5)	C15—C16—C20—O9	−33.3 (5)
O7—N2—C12—C13	−5.9 (6)	C15—C16—C20—N4	145.8 (3)
O8—N2—C12—C11	−5.8 (6)	C17—C16—C20—O9	147.2 (4)
O8—N2—C12—C13	172.6 (4)	C17—C16—C20—N4	−33.7 (5)
Zn1—N3—C15—C16	166.7 (3)	C19—C18—C17—C16	−3.0 (7)
C19—N3—C15—C16	−2.4 (5)	N3—C19—C18—C17	1.0 (7)
C15—N3—C19—C18	1.7 (6)	C23—C22—C21—N5	0.9 (5)
Zn1—N3—C19—C18	−166.4 (3)	C26—C22—C21—N5	−178.9 (3)
Zn1—N5—C21—C22	−163.3 (2)	C21—C22—C23—C24	−3.0 (5)
C25—N5—C21—C22	1.8 (5)	C26—C22—C23—C24	176.8 (3)
Zn1—N5—C25—C24	161.1 (3)	C22—C23—C24—C25	2.3 (6)
C21—N5—C25—C24	−2.6 (5)	N5—C25—C24—C23	0.5 (6)
O1—C1—C2—C3	13.6 (5)	N6—C26—O10—Zn1^ii^	−1.0 (7)
O1—C1—C2—C7	−168.2 (4)	C22—C26—O10—Zn1^ii^	178.5 (3)
O2—C1—C2—C3	−165.0 (4)	O10—C26—C22—C21	36.8 (4)
O2—C1—C2—C7	13.1 (5)	O10—C26—C22—C23	−142.9 (3)
C1—C2—C3—C4	178.5 (4)	N6—C26—C22—C21	−143.6 (3)
C7—C2—C3—C4	0.3 (6)	N6—C26—C22—C23	36.6 (5)

Symmetry codes: (i) −*x*+2, −*y*, −*z*; (ii) −*x*+1, −*y*, −*z*.

### Hydrogen-bond geometry (Å, º)

**Table:**

*D*—H···*A*	*D*—H	H···*A*	*D*···*A*	*D*—H···*A*
N4—H4*B*···O1^iii^	0.86	2.20	2.851 (4)	132
N6—H6*A*···O1^ii^	0.86	1.98	2.814 (4)	164
N6—H6*B*···O9^i^	0.86	2.09	2.880 (4)	153
C15—H15···O6	0.93	2.45	3.079 (4)	125
C19—H19···O3^iv^	0.93	2.57	3.238 (7)	130
C21—H21···O10^ii^	0.93	2.38	3.058 (4)	130

Symmetry codes: (i) −*x*+2, −*y*, −*z*; (ii) −*x*+1, −*y*, −*z*; (iii) −*x*+2, −*y*+1, −*z*+1; (iv) *x*−1, *y*−1, *z*.
